# Fever of unknown origin in a male patient with systemic lupus erythematosus

**DOI:** 10.22088/cjim.8.3.217

**Published:** 2017

**Authors:** Duminda Bandara Basnayake, Thamara Kannangara, Laknath Welagedara, Vindhya Bandara, Janitha Herath

**Affiliations:** 1 Department of Medicine, Teaching Hospital Kandy, Sri lanka.; 2 Department of Haematology, Teaching Hospital Kandy, Sri lanka.

**Keywords:** Fever of unknown origin, Systemic lupus erythematosus, Male, Lymphadenopathy

## Abstract

**Background:**

Systemic lupus erythematosus (SLE) is an inflammatory autoimmune disorder which is uncommon in men. It has a wide variety of clinical presentations.

**Case Report:**

We report a 21-year-old male presented with one month history of fever, loss of appetite, weight loss and reduced hair growth with an examination revealing an oral ulcer, cervical and axillary lymphadenopathy simulating hematological malignancy. Investigations showed pancytopenia, positive anti-nuclear factor and double-stranded DNA, high erythrocyte sedimentation rate with normal C-reactive protein levels and hypocomplementemia. The diagnosis of systemic lupus erythematosus was made and treatment with oral prednisolone conferred a dramatic clinical and biochemical improvement within one week.

**Conclusion:**

In the evaluation of fever of unknown origin, one should be guided by the presenting symptoms and signs of a patient and even though uncommon, SLE is a worthwhile diagnosis to investigate even in a male patient if the clinical picture is suggestive.

**F**ever of unknown origin (FUO) is a disease entity with major diagnostic challenge in clinical medicine. It is defined as fever for 3 weeks or more with a temperature of 38.3ºC or more on at least two occasions in an immunocompetent patient with no evident diagnosis despite thorough history taking, physical examination and primary investigations (1). According to the medical literature, over 200 causes of FUO have been identified. However, they can be subdivided into four main categories: infections, malignancies, non-infectious inflammatory diseases (autoimmune and rheumatic diseases, vasculitis syndromes and granulomatous disorders) and miscellaneous causes (1, 2). One study shows non-infectious inflammatory diseases (NIID) as the second most frequent cause of FUO, of which systemic lupus erythematosus (SLE) accounts for 50% of the cases (3). Even though SLE shows a female preponderance with a female-to-male ratio of 9:1 (4), SLE is one of the diagnoses to be borne in mind when a male patient presents with FUO. We report a case of FUO where the diagnosis turned out as male SLE. 

## Case presentation

A 21-year-old male patient was admitted with 1 month history of intermittent high grade fever. He also complained of weight loss, loss of appetite, generalized body weakness and hair loss. He was not on any long term drug treatment. On clinical examination, he appeared unwell, was febrile at 38.5ºC with mild pallor.

He also had a painful oral ulcer with bilateral cervical and right axillary nontender lymphadenopathy but there was no hepatosplenomegaly or bone tenderness. Other system examinations were unremarkable. Laboratory evaluation revealed normochromic normocytic anaemia (hemoglobin 9.9g/dl, RBC 3.9×10^12^/L), leucopenia (white cell count 2.06×10^9^/L) and thrombocytopenia (platelet 75×10^9^/L)**.** Aspartate transaminase was 64.2 U/L, alanine transaminase 86.7 U/L, GGT 244.4 U/L (normal range 15–55 U/L) and lactate dehydrogenase was 757U/L (230-460 U/L). Alkaline phosphatase, serum albumin and bilirubin levels were normal. Erythrocyte sedimentation rate (ESR) was 55mm in 1^st^ hour and C-reactive protein (CRP) was 3.8mg/L (<6). 

Serum ferritin increased at 1174.36ng/mL (20–159 ng/mL). Coagulation profile was normal. Urea, creatinine and urinalysis were normal. Infection screen for Epstein-Barr, cytomegalovirus, hepatitis B, hepatitis C and HIV viruses was negative. Toxoplasma gondii IgG antibody was positive with negative IgM. Blood and urine cultures were negative. Malarial parasites were not detected. Mantoux test was negative. The autoimmune profile revealed increased levels of double stranded (ds)-DNA antibody 133.3 IU/L (>46.1 IU/L is positive), C3 level was 46.1 mg/dL (normal range 90-180 mg/dL). Anti-nuclear antibody (ANA) was positive. Chest x-ray, electrocardiogram, echocardiogram and abdominal ultrasound were normal. Cervical lymph node biopsy revealed chronic reactive lymphadenopathy without neoplastic lymphoid cell proliferation. There was a reactive bone marrow. The diagnosis of SLE was made according to the American Collage of Rheumatology (ACR) diagnostic criteria, as he fulfilled four criteria including hematological involvement, oral ulcer, positive ANA and ds-DNA antibody. He was started on oral prednisolone 1mg/kg daily treatment. He made a dramatic clinical and biochemical improvement within one week and was discharged. He was reviewed at medical clinic regularly and corticosteroids were tailed off gradually. Currently, three months after the diagnosis, he is being managed with low-dose prednisolone, hydroxychloroquine and osteoporosis prophylaxis.

## Discussion

SLE is a chronic inflammatory autoimmune disorder which involves multiple organs of the body. The onset of the disease occurs between the ages of 16-50 years. Estimated incidence rates range from 1 to 10 per 100,000 person-years and prevalence rates range from 20 to 70 per 100,000 around the world. Nevertheless, the incidence and prevalence rates are nearly 2 to 3 times higher in African and Asian people than in white races (5). SLE is more common in females than males (ratio 9:1) but male gender is associated with a bad prognosis (4, 6). 

Studies show that male SLE is associated with higher incidence of nephropathy, cardiovascular involvement, thrombotic phenomena and anti ds-DNA antibodies which confer a higher mortality rate compared to female patients with SLE (6-8). SLE patients may present with a variety of clinical manifestations resembling many differential diagnoses. Thus, the suspicion of SLE needs to be considered when deducing a diagnosis for FUO. Common manifestations (>30% of cases) in adult SLE include arthritis and/or arthralgia, fever, photosensitivity and malar rash. 

Less common manifestations (10%-30% of cases) are leukocytopenia, Raynaud’s phenomenon, serositis, nephropathy, neurological involvement, oral ulcers, alopecia and thrombocytopenia. There are uncommon features (<10% of cases) such as lymphadenopathy, discoid lesions, sicca syndrome, livedo reticularis, hemolytic anaemia, thrombosis, subacute cutaneous lupus, lung involvement, urticaria and purpura, which patients may present with (9). Our patient had positive findings of fever, leukopenia, oral ulcers, alopecia, thrombocytopenia and lymphadenopathy which guided to the diagnosis**. **According to GLADEL cohort (6) these positive findings are more common in males compared to females, except alopecia. 

According to the ACR diagnostic criteria (Table 1), a patient is classified as having SLE if any 4 or more of 11 criteria are met (10). Nonethess, these criteria may not present in early or limited disease (9). So it should not replace the clinical judgment of the diagnosis. Laboratory evaluation of SLE shows anemia (60%), leucopenia (45%), thrombocytopenia (30%), anti-cardiolipin antibody (25%), proteinuria (30%), hematuria (30%), hypocomplementemia (60%), ANA (95-100%), anti native DNA (50%) and anti-Sm (20%). Usually ESR is elevated but CRP remains normal unless serositis, arthritis or infection exist (11). The complex nature of SLE warrants a multidisciplinary management protocol. Management is primarily decided upon the involvement of major organs. For SLE patients without major organ manifestations, treatment recommendations are antimalarials (cloroquine, hydroxychloroquine) and/or glucocorticoids. Immunosuppressive agents such as azathioprine, mycophenolate mofetil, and methotrexate need to be prescribed if patient is not responding or if low-steroid dose to the acceptable maintenance value brings up relapses (12). 

**Table 1:** ACR diagnostic criteria of SLE

1. Malar rash
2. Discoid rash
3. Photosensitivity
4. Oral ulcers
5. Arthritis
6. Serositis
7. Renal involvement
a) >0.5g/d proteinuria, or
b) ≥3+ dipstick proteinuria, or
c) Cellular casts
8. Neurological disorders
a) Seizures, or
b) Psychosis
9. Hematological disorders
a) Hemolytic anaemia, or
b) Leucopenia(<4000/µl), or
c) Lymphopenia(<1500/µl), or
d) Thrombocytopenia(<10^5^/µl)
10. Immunological abnormalities
a) Antibody to native DNA, or
b) Antibody to Sm, or
c) Antibodies to antiphospholipid antibodies
11. Positive ANA

In conclusion SLE needs to be suspected when analyzing a cause for FUO even in a male patient coming with a compatible clinical picture. After establishing the diagnosis prompt management should follow. In the follow-up process, early detection of organ involvement is crucial as the management protocol differs accordingly and so will the prognosis. 
